# The Ring World: Eversion of Small Double-Stranded Polynucleotide Circlets at the Origin of DNA Double Helix, RNA Polymerization, Triplet Code, Twenty Amino Acids, and Strand Asymmetry

**DOI:** 10.3390/ijms232112915

**Published:** 2022-10-26

**Authors:** Victor Norris, Jacques Demongeot

**Affiliations:** 1 Laboratory of Communication Bactérienne et Stratégies Anti-infectieuses UR4312, Université de Rouen, CEDEX, 76821 Mont-Saint-Aignan, France; 2 Faculty of Medicine, Université Grenoble Alpes, AGEIS EA 7407 Tools for e-Gnosis Medical, 38700 La Tronche, France

**Keywords:** Ring World, Circlet hypothesis, origin of the double-stranded DNA, origin of triplet code

## Abstract

It is not entirely clear why, at some stage in its evolution, terrestrial life adopted double-stranded DNA as the hereditary material. To explain this, we propose that small, double-stranded, polynucleotide circlets have special catalytic properties. We then use this proposal as the basis for a ‘view from here’ that we term the Circlet hypothesis as part of a broader Ring World. To maximize the potential explanatory value of this hypothesis, we speculate boldly about the origins of several of the fundamental characteristics and briefly describe the main methods or treatments applied. The principal prediction of the paper is that the highly constrained, conformational changes will occur preferentially in dsDNA, dsRNA and hybrid RNA-DNA circlets that are below a critical size (e.g., 306 bp) and that these will favor the polymerization of precursors into RNA and DNA. We conclude that the Circlet hypothesis and the Ring World therefore have the attraction of offering the same solution to the fundamental problems probably confronting both the earliest cells and the most recent ones.

## 1. Introduction

There is no consensus about the answers to some of biology’s most fundamental questions. One of these questions is why the hereditary material is in the form of a double-stranded helix in which the DNA strands are wrapped around one another. This structure constrains the movement of the strands and seems completely unsuited to the strand separation that is integral to replication and transcription, and that in modern cells requires the activity of sophisticated helicases and topoisomerases. It might be argued that a linear polymer like a DNA strand naturally forms a spiral and that an inheritance system derived from base pairing of complementary strands must therefore entail a double helix. However, this would be to ignore the possibility of systems based on having a partial coding (so that two independent helices could interact without intertwining), on having several strands coming together (analogous to protofilaments assembling into a microtubule), on having a planar structure (to permit a 2-D coding), etc. An amino acid is an amino group and a carboxyl group plus a highly variable side-chain and, given that five hundred different amino acids have been reported [1], a second problem is then why biological systems code for so few amino acids (twenty classical ones plus selenocysteine and pyrrolysine) when so many seem available [2]. It is tempting to suspect that this limited number is somehow related to a third problem, namely, why there is a triplet code.

In the case of the RNA World scenario, other fundamental problems have been raised though possible solutions have been proposed, see below [3], and though substantial progress has been made recently with the generation of poly-ribonucleotides hundreds of subunits long in the presence of rock glasses [4]. For example, the hypothesis that a prebiotic system existed in which a ribozyme alternates between acting as a template for replication and acting as a catalyst has the following problems: firstly, the folded state of the RNA that is required for catalysis may be largely incompatible with the unfolded state required for replication; secondly, a successful template-directed RNA synthesis yields a duplex that may prevent both the old and new strands from serving as templates for another round of replication (known as the strand inhibition problem); thirdly, a replicated RNA in its duplex state would have the thermodynamically difficult task, at least in water, of going from the duplex state to the single-stranded state needed for catalysis.

**Figure 1:**
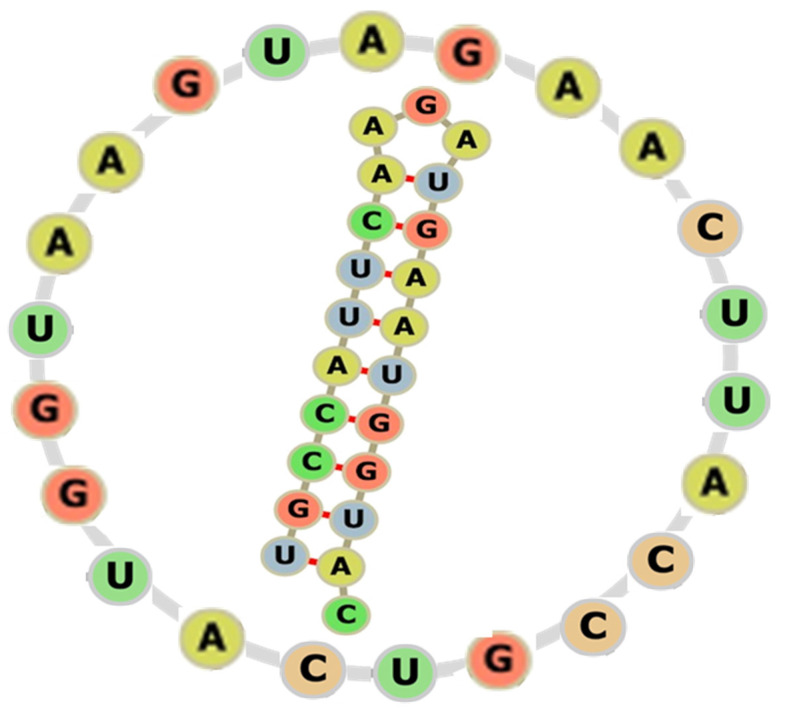
Ring and hairpin conformation of the RNA circlet AL.

At an early stage in the origins of life, interfaces between lipid domains have been suggested as catalysts of the polymerization of nucleic acids [5] and, given the circularity of such domains, their catalysis of polynucleotides into rings seems a reasonable possibility. In line with this, the evidence for a role for lipids in catalysis continues to grow, especially when solutions are subject to wet-dry cycles [6]. Such cycles can generate ring-like structures of RNA down to 10 nm in diameter, some of which can pair with one another to make complex structures [7]. These rings are reminiscent of viroids, which are plant pathogens made out of a small, single-stranded RNA circle, which can possess catalytic properties, and which have been considered to be ‘molecular fossils’ [8]. Indeed, there is an increasing interest in the idea that a circular RNA could have been important in the origins of the hereditary material [9]. One approach is to determine which sequence of an original ring(s) was most likely to have encoded twenty amino acids plus start and stop codons. A set of 25 theoretical, minimal, single-stranded RNA rings comprising 22 nucleotides were designed in silico to code for each amino acid by using the three reading frames and to form stem-loop hairpins [10]. These primordial rings turned out to possess characteristics of the translational machinery of modern cells and independent secondary structure analyses showed that most of these rings resemble the predicted proto-tRNAs [10]. In the eventuality of a single primordial ring at the root of the code, the “alpha” or “AL-ring” was considered the best candidate on the basis of its evolutionary relationship to the other candidate rings (Figure 1).

Here, we propose the Circlet hypothesis as a subset of a broader Ring World approach to the origins of RNA and DNA. In this hypothesis, polynucleotide rings played a fundamental role in the origins of life. In particular, double-stranded polynucleotide rings averaging 22 bp, that we term circlets, acted as catalysts of many reactions and, in particular, acted to catalyze the polymerization of nucleotides into double-stranded copies of these circlets. To maximize the use of this hypothesis in the conception of experiments, we explore it by speculating freely: we relate it to the triplet code and the limited number of amino acids used by living systems, show how it may solve problems with the RNA World, extend it to explain strand asymmetry, suggest it connects replication and cell division mechanistically, and present evidence supporting it. Finally, we situate the hypothesis between the prebiotic ecology of ‘composomes’ (physically interacting ensembles of molecules exhibiting compositional inheritance) and the emergence of cells in which metabolism and replication are coupled and coordinated [11,12,13].

## 2. The Circlet Hypothesis

**Figure 2:**
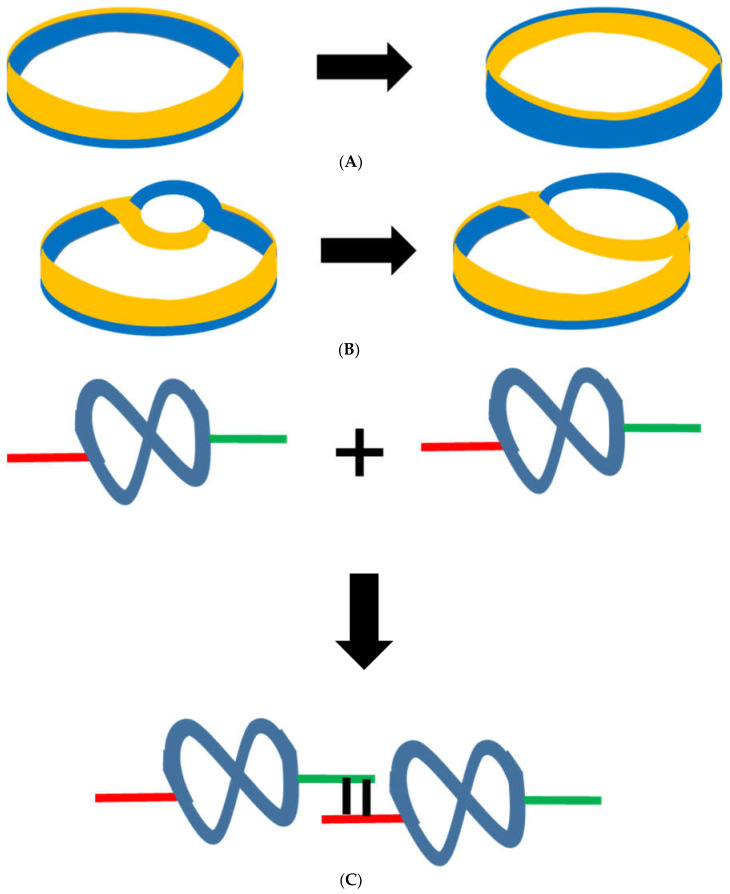
Eversion hypothesis. (**A**) Eversion of a ring made from two ribbons takes the blue ribbon from the outside of the circle to the inside and then back to the outside to complete the cycle. (**B**) Eversion of the ring creates a moving and varying distortion between the ribbons that can catalyze reactions. (**C**) Distortions including base-flipping (red and green lines) and subsequent interaction between these bases (black lines) leads to circlets associating with one another (and other molecules) to form filaments or networks.

The reason the double helix of DNA has been selected is that rings or circlets of partially or completely double-stranded RNA, DNA and RNA/DNA hybrids were created during life’s origins with catalytic activities that were greatest and most effective for the smallest circlets. This catalysis occurred because of the dynamic variations of the conformations adopted by these circlets; these conformations depended on distortions of the bonds between the base pairs leading to the separation of the strands. Reproducible cycles of catalytic conformations were generated as the circlet underwent eversion about the curved long axis of the double helix as, for example, a base on the inside of the circlet moved to the outside and vice versa (Figure 2). Small thermal fluctuations everted the circlet slightly back and forth along the catalytic trajectory (the meaning of eversion is shown in Figure 2) with the consequence that polymerization of two or, more probably, three ribonucleotides or nucleotides was catalyzed if they hybridized with the distorted region of the circlet, thereby giving rise to the triplet code. The double-helical, closed structure of circlets created constraints that favored this polymerization and, if the circlet continued to undergo eversion in the same direction, this polymerization continued to create longer stretches of both RNA and single-stranded DNA; such processive polymerization was favored by the continuing association of the newly synthesized polymer with the template, which acted in a ratchet-like manner. The nature of the eversion of the small circlet exposed bases on both strands and permitted replication to be either uni- or bi-directional. When the direction of eversion reversed, the newly synthesized strand was detached. Some of these strands bound amino acids and lipids and thereby concentrated cellular precursors around the catalytic circlet; this concentration also favored peptide synthesis.

The hypothesis has seven corollaries. The first corollary is that, during the origins of life, many circlets formed with different sequences and of different sizes; each circlet was capable of weakly catalyzing a different reaction or set of reactions.

The second corollary is that the circlets were physically associated with a wide diversity of the myriad simple, universal, molecules and ions present at the origins of life. Such associations greatly increased the catalytic range and efficacy of the circlets.

The third corollary is that the asymmetry of the strands was important and resulted in different strands in the same circlet conferring different reactions. In some circlets, one strand catalyzed the synthesis of peptides that adopted one structure whilst the other strand catalyzed the synthesis of peptides that adopted a different structure (or no structure at all); for example, one strand catalyzed the synthesis of the alpha-helical form whilst the other strand catalyzed the synthesis of the beta-sheet form. Moreover, one strand catalyzed the synthesis of RNA better than the other, hence facilitating differentiation (see below). The fact that part of a circlet could hybridize with another part (as in hair-pin formation) led to a dynamic equilibrium between three conformations corresponding to the ds-circlet, a denatured state and a single strand folded on itself to trigger replication.

The fourth corollary is that some dsDNA circlets favored the bidirectional polymerization of nucleotides (i.e., copying both strands) to yield dsDNA as well as the uni-directional polymerization of ribonucleotides (i.e., copying only one strand) to yield ssRNA.

The fifth corollary is that regions of homology exposed by the distortions of these circlets allowed them to associate in networks (Figure 2). The ensemble of these networks of circlets catalyzed a large number of reactions (some of which are still found in modern cells) and, reciprocally, the products of these reactions affected the conformational changes of the circlets so as to guide the catalysis of circlet replication and other reactions. This resulted in catalytic closure.

**Figure 3:**
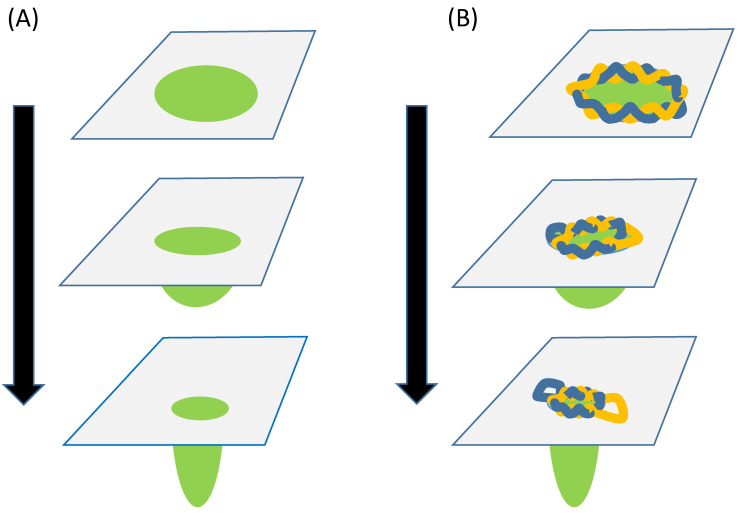
Relationship between membrane invagination and circlet conformation (**A**) The energy associated with the interface of a domain in a planar lipid membrane is reduced by the invagination of the domain lipids into the underlying medium. (**B**) As this invagination occurs, a circlet that binds to the interface undergoes a progressive eversion along with conformational changes. Green, membrane domain; yellow and blue, DNA strands.

The sixth corollary is that genome replication and cell division were coordinated by circlets (Figure 3). This coordination comprised: (i) a circlet binding to the interface between a lipid domain and the rest of a membrane on the inside or on the outside of a vesicle; (ii) this domain undergoing an invagination or a budding; (iii) a continuing—catalytic—eversion of the circlet accompanying and being modified by the process of invagination or budding; and (iv) a modification by the circlet of the dynamics of the invagination or budding process (e.g., slowing the progress of such processes and limiting them to large circlets).

The seventh corollary is the possibility that the genes coding for enzymes in modern genomes may contain sequences that bind the inducers of the expression of the gene and even bind the enzyme’s substrates; such sequences would be ‘fossils’ of original circlets.

## 3. Evidence

### 3.1. Creation of Small RNA and DNA Circles

The smallest circles that can readily exist are below the persistence lengths of the polymers, which depend on temperature and on the nature and concentration of ions (note that one nucleotide unit measures 0.33 nm). In the case of RNA, wet-dry cycles can produce circles containing 50 nucleotides and probably less [7]; the intramolecular cyclization of ssRNA by T4 RNA ligase 2 is reported to occur only when this RNA has certain structures and exceeds a critical size—around 15 nucleotides—with 23 nucleotides being better still [14,15]. The existence of an ancestral, minimal ssRNA circle with a specific sequence—the AL sequence—containing only 22 bases (Figure 1) and with long sub-sequences resembling sequences from modern tRNAs and 5S rRNA is predicted to have arisen at the origins of life [16] (see Supplementary Table S1); circular RNA, which plays an important regulatory role in modern cells [17,18], exists in a wide range of sizes that go down to 63 nucleotides in bovine skeletal muscle [19]; an ssRNA circle containing 21 nucleotides has been used as the basis for a dsRNA circle [20]. In the case of ssDNA, persistence lengths of 4.6 nm [21] and 1.98 ± 0.72 nm [22] have been reported, whilst ssDNA circles of 20 nucleotides have been made using freezing, lyophilizing and rewetting [23]. In the case of dsDNA, persistence lengths of 67 bp have been obtained [24]; moreover, thermal fluctuations result in a 4° bend per bp, which can increase to 20° when cations are transiently associated with the DNA [25]. In the case of DNA circles containing both double-stranded and single-stranded regions, circles of 64 bp have been made [26], which is likely to be over the lower limit since imperfect duplexes can have mismatches, bulge loops, internal nicks and single-stranded gaps that serve as hinges [26]. In the case of RNA-DNA hybrids, which also play important roles in modern cells (for references see [27,28], persistence lengths of 49 to 63 nm have been obtained depending on the NaCl concentration [29]. It may be that the minimum size of a double-stranded polynucleotide circlet depends on the way the circlet is produced with the persistence length of the linear double-stranded polynucleotide being largely irrelevant if the single-stranded circle is produced first. In such a scenario, it should be noted that the persistence lengths of ssRNA and ssDNA depend not only on the valence and concentration of ions but also on their proximity to double-stranded duplexes [30]; moreover, the flexibility needed for circularization is sequence-specific [31].

### 3.2. Topological Strains

It can be useful to consider the topology of circular DNA as that of a Möbius strip, which could favor ‘defects’ and topological discontinuities (the passage from cyclic dsDNA to ssDNA produces a ring of double length) [32]. Such results of topological strains are inversely related to the size of nucleotide circles. In studying dsDNA circles containing cytosine-rich sequences that can fold into tetraplex structures known as i-motifs, it was found that topological strain increased as the size of the circle decreased from 96 to 75 bp and that this affected the folding of the motif [33]. In supercoiled dsDNA circles of 339 bp, bends of 140° have been observed in stretches of 16 bp along with disruptions to base-stacking and complementary base pairing [34]. In smaller dsDNA circles, disruptions of the regular DNA structure by bending deformation occurred when the average angle between adjacent base pairs approached 6° (in the range of the average amplitude of the 6–7° angles resulting from thermal fluctuations); importantly, such disruptions resulted from DNA bending alone in 64–66 bp circles but not in bigger circles [26].

The linking number, i.e., the number of times the two strands of closed-circular DNA are linked, Lk, is the sum of the twist (the number of times one strand of DNA turns around the other strand) and writhe (the number of times the double helix turns around itself); if a 1050 bp dsDNA circle were in the relaxed form, it would have an Lk0 of 100 (1050 base pairs divided by 10.5, the number of base pairs per turn) [35]. Supercoiling is the result of departures from Lk0, which is termed the linking difference, ΔLk. Hence, disruptions of structure in dsDNA circles have been attributed to the torsional stress that results when ΔLk is less than zero; for example, a circle formed from a fragment of 100 bp, which has a semi-integer number of helix turns, yields a topoisomer with an ΔLk of –0.5 (the topoisomer with an ΔLk of +0.5 was not observed) that can be attacked by a nuclease [36]. Similarly, defects were observed in negatively-supercoiled circles of dsDNA of 251 and 339 kb with ΔLk = −1 (but not in the relaxed topoisomer) and most of these defects were denaturation bubbles, where two or more base pairs are flipped out of the duplex, which allows a 180° turn within a single helical turn [34]. Equivalent bending and supercoiling-induced deformations have been obtained with circles between around 60 and 100 bp [37,38,39]. Finally, a molecular dynamics study of 30 bp compared to 180 bp dsDNA circles revealed differences in conformational dynamics consistent with eversion in the former circles [40].

### 3.3. Catalysis by Polynucleotides

RNA and DNA have long been known to be capable of catalyzing certain reactions, particularly when they function as metalloenzymes; this is not only because divalent cations can be cofactors in reactions involving RNA and DNA phosphoester transfer and hydrolysis (Mg^2+^, for example, can serve as a Lewis acid) but also because they can participate in folding by helping pack phosphodiester chains (for references see [41]). Significantly, DNA enzymes can catalyse the formation of a 3’-phosphodiester linkage between DNA oligonucleotides [42]. The problematic transition from a newly synthesized ribozyme sequence in its inactive duplex state to its active folded state has been shown to be facilitated by a viscous solvent and a fluctuating environment [43].

In the case of circular RNA and DNA, conformational changes are linked to catalysis. Supercoiled circles of DNA undergo conformational changes that lead to the binding of external substrates such as another DNA strand [34]. The processivity of polymerization of RNA or DNA that depends on a continued eversion of the circlet could have been achieved if the newly replicated strand acted as a ratchet to prevent the circlet reversing the direction of eversion. Such processivity could be favored by a local environment of amino acids loosely bound to the 2–3 nucleotides exposed by the eversion of the circlet [43,44]. Different sequences of nucleotides would undergo different conformational dynamics, as modulated by the binding of the nascent peptide itself to the circlet, which could help generate different sequences of peptides. Processivity could also result from the binding of a circlet to the interface between a lipid domain and the rest of a membrane on the inside or on the outside of a vesicle followed by the invagination or budding of this domain. Such invagination/budding could occur to minimize the line energy associated with the interface [45] (which increases with the osmotic pressure [46]) or for other reasons [47].

Recently, rolling circle replication has been achieved using a 36 nucleotides single stranded RNA circle as initial template, a 9 base primer, and an RNA-polymerizing ribozyme supplied with trinucleotide triphosphates (rather than the usual DNA polymerase supplied with deoxyribonucleoside triphosphates); this replication continued when the circle became double-stranded RNA, which required duplex invasion and displacement of the primer/product strand, and, significantly, this replication was facilitated by the strain accumulating in the nascent dsRNA segment [46].

The stereochemical matching of amino acids and nucleotide triplets/anti-triplets gives insight into how the genetic code originated [47,48]. There is a predicted order in which amino acids were assigned to codons during the development of the genetic code [49]; in this order, amino acids that were integrated early into the genetic code tend to bind their codons whilst those that were integrated late tend to bind their anticodons [48]. The idea that translation was initially based on codon-amino acid affinities before it was mediated by tRNA adaptors may explain the direct interactions between mRNAs and their cognate proteins in modern cells [50]. Intriguingly, the sequences of the 25 primordial rings turn out to maximize the affinity between their nucleotide triplets and the corresponding cognate amino acids. Moreover, the calculated interactions between these RNA rings and their cognate peptides are strongest for ancient RNA rings and weakest for recent RNA rings, consistent with their putative role in pre-tRNA- to tRNA-mediated translation [51,52,53].

In the case of the peptide bond, it should be noted that catalysis of peptide bonding has been obtained with single-stranded RNAs [54], that two RNA fragments from the peptidyl transfer center of the ribosome are able to catalyze the synthesis of a 9-mer polylysine [55], and that circular nucleic acids containing a ribozyme or DNAzyme or a DNA or RNA aptamer are now being used in topology-controlled molecular devices [56]. Recently, it has also been proposed that that “a universal internal small RNA pocket-like segment” has played the role of a proto-ribosome, and is still embedded in the contemporary ribosome [57].

Finally, in the case of the possible binding of small molecules—and even of subsequent catalysis by entire chromosomes in vivo—there are many drugs that regulate DNA conformation. These drugs have been classed into those that intercalate (e.g., carbazole, anthracycline and acridine) or bind to the major or minor grooves (e.g., pluramycins, aflatoxins, azinomycins, distamycin A, DAPI and Hoechst 33 258) or bind covalently (e.g., cyclophosphamide, mitomycin C and the hydrazines) [58]. Such binding can be sensitive to the conformational state of the DNA and, for example, the intercalator, psoralen, binds duplex DNA at a rate directly proportional to helical tension such that this binding is strongest where the DNA is underwound [59]. Conformational changes and binding are fundamental to catalysis. Encouragingly, a recent modelling approach predicts several xenobiotic metabolites likely to bind DNA in vivo [60]; these predictions therefore increase the plausibility of the binding to DNA of many natural intracellular metabolites.

### 3.4. Strand Asymmetry

The putative role of asymmetry within the circlets may be reflected in modern microRNAs, which are single-stranded, contain around 22 nucleotides and can fold back on themselves to form hairpin structures (note here the importance of denaturation of double-strands [61]). Indeed, another possible solution to the RNA World problem of reconciling the templating ability needed for replication and the stable folding needed for ribozyme activity is to exploit G:U wobble pairing in RNA since, unlike Watson-Crick base pairs, wobble pairs make a big contribution to the energetic stability of the folded structure but only a small contribution (if any) to the stability of the folded reverse complement; simulation of RNA folding has shown that this could be exploited if each strand were to have a different role [8]. In line with this, viroid RNA sequences, which are possible relics (or rediscoveries) of an RNA World [62], also show significant asymmetry in folding energy between the infectious (+) and template (-) strands due to G:U pairing [63]. In the RNA World, strand asymmetry has also been proposed as the basis for the differentiation between templates and catalysts [64] and in silico experiments on populations of protocells containing self-replicating, catalytic genomes have shown that the protocells evolve towards maximizing the numbers and catalytic activities of one strand whilst decreasing the numbers and catalytic activities of the other strand [65]. In modern cells, strand asymmetry is considered as important in generating phenotypic heterogeneity in both prokaryotes [66,67,68] and eukaryotes [69,70].

### 3.5. Polynucleotide Networks

In synthetic chemistry, DNA homologies have been used to link biochemical networks and, for example, two networks have been coupled to mimic the photosynthetic and dark-operating machinery of plant cells [71]. In experiments that generated RNA rings via wet-dry cycles, complex structures were also generated in the form of short polymer attachments and pairing of rings [7]. In considering the formation of polynucleotide networks, it should be noted that nucleic acid fragments with specific sequences can adopt a parallel orientation that depends on non-canonical base-pairing [72].

### 3.6. Lipid Interactions

The Calculations done in [5] are consistent with the idea that the interface between a lipid domain and the rest of the membrane could concentrate, align and orientate nucleic acids and thereby promote their polymerization and, in a similar way, those of amino acids. It is conceivable that catalytic conformations could result from the formation of an oligonucleotide ring in a droplet on a surface at the ‘circular’ interface of water, air and surface followed by the shrinking of that droplet. In the case of droplets on glass, most of the material forms a ring around the edge of the original droplet (David Deamer, personal communication). Droplets might also undergo different sorts of phase separation (gaseous, liquid-expanded, liquid-condensed) as the surface was compressed by the droplet shrinking; this could lead to the sorting of the lipids into different domains with circular boundaries to putatively bind nucleotides and favor the formation of oligonucleotides; those oligonucleotides that remained associated with the membrane would be deformed as the membrane itself continued to be deformed. Certainly, phospholipid vesicles can undergo many structural changes such as fusing into multilamellar films upon drying, thereby trapping solutes such as nucleotides between the lipid layers [73,74]. Significantly, clay particles can catalyze the polymerization by the active forms of amino acids [75].

## 4. Testing the Hypothesis

### 4.1. The Principal Prediction

The main prediction is that the highly constrained, conformational changes will occur preferentially in dsDNA, dsRNA and hybrid RNA-DNA circlets that are below a critical size (e.g., 30 bp), and that these will favor the polymerization of precursors into RNA and DNA. Polyacrylamide gel electrophoresis and mass spectrometry could be used to detect such polymerization while Molecular Dynamics (MD) and Atomic Force Microscopy (AFM) could be used to model and confirm the conformational changes responsible. The simultaneous binding of two or three nucleotides to one position in a strand of the circlet might be detected via MD and AFM, perhaps complemented by Secondary Ion Mass Spectrometry (SIMS) using different stable isotopes [76].

### 4.2. A Limited Prediction

A limited prediction is that the polymerization of nucleic acids will be particularly pronounced in a 22 bp circlet with the sequence of the AL-ring [16], given that bendability is a function of sequence [77]. A dsDNA circlet based on the AL sequence will, during wet-dry cycles, catalyze the polymerization of nucleotides to yield two daughter dsDNA circlets per round of replication but only catalyze the polymerization of ribonucleotides to yield one ssRNA corresponding to one of the strands.

### 4.3. A Specific Prediction

Given the importance attributed in the RNA world to a role for some peptide-free rRNA structure in translation, a specific prediction is that homologies should exist between the sequences of the AL-ring and its complement and modern rRNA (just as they do between the AL-ring and tRNA [53]. Such homologies actually exist; indeed, the most pentanucleotides in common with the AL-ring occur in the genes encoding the 5S proteins of Archaea, which are believed to be the oldest prokaryotes and which have a metabolism (like methanogenesis) that reflects Earth’s primitive atmosphere (Supplementary Table S1).

### 4.4. A More General Prediction

A more general prediction is that diverse populations of small circlets would catalyze a subset of reactions involving small molecules present in modern cells. This would require mixing the circlets with these molecules and detecting any changes in the mixture resulting from catalysis using mass spectrometry. A related prediction is that there would be a relationship between the nucleotide sequence of a circlet catalyzing a particular reaction and the amino acid sequence of the active site of the modern enzyme catalyzing the same reaction.

### 4.5. A Highly Speculative Prediction

A highly speculative prediction is that a diverse population of small circlets plus simple sources of carbon, nitrogen, oxygen, hydrogen and other elements would achieve catalytic closure and hence be able to grow if supplied with simple precursors.

### 4.6. The Assembly of Circlets

The assembly of circlets into linear or branched filaments and the nature of the links between these circlets (Figure 2C) could be tested using AFM, along with the combination of DNA combing, isotope-labelling and SIMS [77,78].

## 5. Discussion

Constraints are central to biology. Kauffman has argued that a circular relationship between work and constraints allows self-determination, in the form of a “work–constraint cycle” like a Carnot engine (plus a Watt regulator to provide negative feedback) but with the difference that the constraints are produced and maintained by the system itself [79,80]. In the Circlet hypothesis, it is the constraints on the conformational dynamics of small double-stranded circlets that are responsible for the very properties leading to life originated in its terrestrial form. These properties include the potential to catalyze a wide range of reactions and, in particular, the polymerization of RNA and DNA using triplets of ribonucleotides and nucleotides.

The Circlet hypothesis and, more generally, the Ring World, fit in with the ‘prebiotic ecology’ [12] in which a flux of abiotic creation and destruction of molecules led to the selective accumulation of molecules that interacted via molecular complementarity [81,82] to form composomes responsible for compositional inheritance [11]; in such composomes, DNA’s elastic properties could have helped maintain structural integrity [83], while each small, everting circlet could have become a different catalytic engine at the heart of a different composome.

In addition, the flipping out of bases [84] and their subsequent interactions could have led to the association of circlets with one another in extended networks (Figure 3). The eversion hypothesis also fits in with the idea that hybrid RNA-DNA molecules preceded homogeneous RNA and DNA [85]. It is therefore encouraging that RNA-DNA hybrids can catalyze the polymerization of both RNA and DNA [86].

By proposing that the units of evolution were—or contained, in the case of composomes—small, catalytic, double-stranded polynucleotide circlets in which each strand has a different role, the Circlet hypothesis helps solve some of the problems associated with the RNA World. These include the problem of reconciling the folded versus unfolded states needed for a molecule to act as both catalyst and template, and the problem of a duplex forming that prevents further replication. Solutions to these problems have been proposed based on strand asymmetry [8,62,63,64,65]. A different problem that confronts modern cells—and that may have confronted the first cells too—is that of generating phenotypic heterogeneity. Strand asymmetry has been proposed as a solution [66,67,68,69,70] here as well.

The Circlet hypothesis and the Ring World therefore have the attraction of offering the same solution to the fundamental problems that probably both confronted the earliest cells and continue to confront the most recent ones.

In proposing these hypotheses, we have chosen to include here their most speculative predictions because of the possible insights they provide. One example is in going beyond positing that double-stranded circlets catalyzed their own synthesis to positing that, as parts of composomes containing subsets of thousands of small, different, universal molecules such as lipids and ions [87], these circlets also catalyzed a wide variety of the reactions found in modern metabolism.

A second example is in positing that a circlet was associated with the interface between a lipid domain and the rest of the membrane; such interfaces have been proposed to play a role in (i) the polymerization of nucleic acids and amino acids [5]; and (ii) the budding and invagination processes that are central to cell division [88,89]. These interactions would have been two-way with the domain and its interface altering the conformational dynamics of the circlet and, reciprocally, the circlet altering the dynamics of the domain (e.g., so as to advance, position or retard the process of division).

## 6. Conclusions

From a theoretical perspective, the degree of speculation of the present article allows the circlet hypothesis to be related to the hypothesis of composomal inheritance and to help explain the transition from the prebiotic ecology to the world of coding, genome replication and cell division. From the experimental perspective, this degree of speculation may help the hypothesis to stimulate many different types of experiments, the most extreme of which would be that (1) different small everting circlets would catalyze (albeit weakly) different subsets of the reactions present in modern cells; (2) each strand in a given circlet would have a separate homology at the nucleotide or amino acid level with a modern enzyme; and (3) a few of the nucleotide sequences coding for enzymes in modern genomes may still contain small ‘fossil’ sequences that bind the enzyme’s substrates and even perform a residual catalysis themselves. It has therefore not escaped our attention that such catalytic circlets would have implications for biotechnology.

## Data Availability

Not applicable.

## Supplementary Materials

The supporting information can be downloaded at: https://www.mdpi.com/article/10.3390/ijms232112915/s1.
