# Supplementary material for: The Ring World: Eversion of Small Double-Stranded Polynucleotide Circlets at the Origin of DNA Double Helix, RNA Polymerization, Triplet Code, Twenty Amino Acids, and Strand Asymmetry
Source: Int J Mol Sci. 2022 Oct 26;23(21):12915. doi: 10.3390/ijms232112915 (PMC9657125; doi:10.3390/ijms232112915)
Supplement: Supplementary file 1 [file ijms-23-12915-s001.zip › ijms-1928154-supplementary.pdf]

Table S1. Homologies between the alpha ring and its complementary strand antiAL and 5S rRNA sequences from Archaea. Inside the 5S sequences, we represent in red the pentanucleotides (in blue if they are adjacent) common with AL, and in green if they are common with antiAL. The expected value of the percentage of the pentamers coming from AL or antiAL rings in the 5S structure is equal to 2.1% ± 2.3% (1-sided 0.95-confidence interval). All observed 5S sequences have more than 4.4% of pentamers from AL and only 3% from anti-AL, where:

AL: ATGGTACTGCCATTCAAGATGA **antiAL: TCATCTTGAATGGCAGTACCAT**

>NZ_JAGGMO010000007.1 Methanococcus voltae strain DSM 14649 Ga0466840_07
TACGGCGGTCATAGCGGAGGTGTTCCATCCGATC**CCATTC**CGATCTCGGAAATTAAGCCCTCCAGC
GATTTCTTAA**GTACTGCCATATGGT**GGGAA**CAAGATGA**CG**CTGCC**GATC

AL-Pentamer number	5S sequence length	Percentage of AL pentamers
14	111	**12.6**

TACGGCGGTCATAGCGGAGGTGTTCCATCCGATC**CCATTC**CGATCTCGGAAATTAAGCCCTCCAGC
GATTTCTTA**AGTAC**TGCCATATGGTGGGAACAAGATGACGCTGCCGATC

AntiAL-pentamer number **3**

>A00001 Methanococcus voltae
TGATACGGCGGTCATAGCGGAGGTGTCCCATCCGATC**CCATTC**CGATCTCGGAAATTAAGCCCTCCA
GCGATTTCTTAA**GTACTGCCATATGGT**GGGAA**CAAGATGA**CG**CTGCC**GATCAC

14 116 **12.1**

TGATACGGCGGTCATAGCGGAGGTGTCCCATCCGATC**CCATTC**CGATCTCGGAAATTAAGCCCTCCA
GCGATTTCTTA**AGTAC**TGCCATATGGTGGGAACAAGATGACGCTGCCGATCAC

3

>NZ_JAGGMO010000007.1 Methanococcus voltae strain DSM 14649 Ga0466840_07
CATAACGGTCATAGCGGAGGTGTACATCCGATC**CCATTC**CGATCTCGGAAATTAAGCCCTCCAGCG
ATTTCTTAA**GTACTGCCATATGGT**GGGAA**CAAGATGA**CGCTGTTAGTC

13 109 **11.9**

CATAACGGTCATAGCGGAGGTGTACATCCGATC**CCATTC**CGATCTCGGAAATTAAGCCCTCCAGCG
ATTTCTTA**AGTAC**TGCCATATGGTGGGAACAAGATGACGCTGTTAGTC

3

>NR_075764.1 Methanococcus voltae A3 strain A3 5S ribosomal RNA, complete sequence
TAACGGTCATAGCGGAGGTGTACATCCGATC**CCATTC**CGATCTCGGAAATTAAGCCCTCCAGCGAT
TTCTTAA**GTACTGCCATATGGT**GGGAA**CAAGATGA**CGCTGTTAGTCA

13 109 **11.9**

TAACGGTCATAGCGGAGGTGTACATCCGATC**CCATTC**CGATCTCGGAAATTAAGCCCTCCAGCGAT
TTCTTA**AGTAC**TGCCATATGGTGGGAACAAGATGACGCTGTTAGTCA

3

>NZ_AJJU01000016.1 Imtechella halotolerans K1 Contig16, whole genome shotgun sequence
TAAGGTGGTTATAGC**AATGG**GGCTCACCTCTTACCTTTCCGAACAGAGTAGTTAAGC**CCATT**AGCG
C**AGATGGTACTGCCAT**CCGGTGGGAGAGTATGTCGCCGCCTTCT

12 106 **11.3**

TAAGGTGGTTATAGC**AATGG**GGCTCACCTCTTACCTTTCCGAACAGAGTAGTTAAGC**CCATT**AGCG
CAGATGGTACTGCCATCCGGTGGGAGAGTATGTCGCCGCCTTCT

2

>JMFY01000026.1 Clostridium sp. HMP27 Clostridium-HMP27_contig_26, whole genome shotgun sequence
TCTGGTGATTATGGCTTGAAGGTAACACCCGTAC**CCATTC**CGAACACGAAGGTTAAGC**TTCAA**AG
CGCC**AATGGTACTGCC**GGGGAGGCCCGGTGGGAGAGTAGGTCGT**TGCCA**GAT

12 113 **10.6**

TCTGGTGATT**ATGGCTTGAA**GGTAACACCC**GTACCCATTC**CGAACACGAAGGTTAAGCTTCAAAG
CGCC**AATGG**TACTGCCGGGGAGGCCCGGTGGGAGAGTAGGTCGTTGCCAGAT

7

>NR_075496.1 Methanococcus aeolicus Nankai-3 strain Nankai-3 5S ribosomal RNA, complete sequence

TAACGGTCATAGCGAAGGTGTTATATCCGATC**CCATTC**CGATCTCGGAAA**TCAAG**CCCTTCAGCG
ATTCCCTAA**GTACTGCCATTC**GGTGGAAACAGGGAGACGCTGTTAGT

11 108 **10.2**

TAACGGTCATAGCGAAGGTGTTATATCCGATC**CCATTC**CGATCTCGGAAATCAAGCCCTTCAGCGA
TTCCCTA**AGTAC**TG**CCATTC**GGTGGAAACAGGGAGACGCTGTTAGT

5

>NZ_KB907848.1 Hymenobacter aerophilus DSM 13606 H145DRAFT_scaffold00049.49
AATGGTGGCTTTAGCCCGGGTGTTCACCTCTTC**CCATTC**CGAACAGAGTCGTTAAGCCCCGGAGCG
CCT**ATGGTACTGCC**TTCACCGGTGGGAGAGTCGGTCGCCGCCAACC

11 108 **10.2**

AATGGTGGCTTTAGCCCGGGTGTTCACCTCTTC**CCATTC**CGAACAGAGTCGTTAAGCCCCGGAGCG
CCTATGGTACTGCCTTCACCGGTGGGAGAGTCGGTCGCCGCCAACC

3

>MGWI01000006.1 Flavobacteria bacterium GWF1_32_7 gwf1_scaffold_15, whole genome shotgun sequence
GGGCTCACCTCTTC**CCATTC**CGAACAGAGAAGTTAAGCCCGATTGCGC**AGATGGTACTGC**AGTTA
TGTGGGAGAGTATGTCGCCGCCTTTC

9 87 **10.3**

GGGCTCACCTCTTC**CCATTC**CGAACAGAGAAGTTAAGCCCGATTGCGCAGATGGTACT**GCAGT**TA
TGTGGGAGAGTATGTCGCCGCCTTTC

3

>NR_103393.1 Thermobifida fusca YX strain YX 5S ribosomal RNA, complete sequence
TACGGCGGTCATAGCGGGCGGGGTCCACCCGGTCCCATTCCGAACCCGGTCGTTAAGCCGCCCAGC
GCCGATGGTACTGCCCTGTGGTGGGGTGGGAGAGTAGGACACCGCCGTAC

9 112 **8**

TACGGCGGTCATAGCGGGCGGGGTCCACCCGGTC**CCATTC**CGAACCCGGTCGTTAAGCCGCCCAG
CGCCGATGGTACTGCCCTGTGGTGGGGTGGGAGAGTAGGACACCGCCGTAC

2

>NZ_BCRL01000048.1 Rhodococcus hoagii NBRC 101255 = C 7 strain NBRC 101255, whole genome
TACGGCG**GCCAT**AGCGGAGGGGAAACGCCCGGTC**CCATTC**CGAACCCGGAAGCTAAGCCCTCCA
GCGCCG**ATGGTACTGC**ACTCGACAGGGTGTGGGAGAGTAGGACACCGCCGAAC

9 113 **8**

TACGGCGGCCATAGCGGAGGGGAAACGCCCGGTC**CCATTC**CGAACCCGGAAGCTAAGCCCTCCAG
CGCCGATGGTACTGCACTCGACAGGGTGTGGGAGAGTAGGACACCGCCGAAC

2

>NR_075410.1 Rhodococcus jostii RHA1 strain RHA1 5S ribosomal RNA, complete sequence
AGTTACGGCG**GCCAT**AGCGGAGGGGAAACGCCCGGTC**CCATTC**CGAACCCGGAAGCTAAGCCCT
CCAGCGCCG**ATGGTACTGC**ACCCGACAGGGTGTGGGAGAGTAGGACACCGCCGAAC

9 116 **7.8**

AGTTACGGCGGCCATAGCGGAGGGGAAACGCCCGGTC**CCATTC**CGAACCCGGAAGCTAAGCCCTC
CAGCGCCGATGGTACTGCACCCGACAGGGTGTGGGAGAGTAGGACACCGCCGAAC

2

> NR_075246.1 Rhodococcus opacus B4 strain B4 5S ribosomal RNA, complete sequence
GTTACGGCG**GCCAT**AGCGGAGGGGAAACGCCCGGTC**CCATTC**CGAACCCGGAAGCTAAGCCCTCC
AGCGCCG**ATGGTACTGC**ACCCGACAGGGTGTGGGAGAGTAGGACACCGCCGAACA

9 116 **7.8**

GTTACGGCGGCCATAGCGGAGGGGAAACGCCCGGTC**CCATTC**CGAACCCGGAAGCTAAGCCCTCC
AGCGCCGATGGTACTGCACCCGACAGGGTGTGGGAGAGTAGGACACCGCCGAACA

2

> GenBank X55254.1 R. rhodochrous 5S rRNA

GTTACGGCG**GCCAT**AGCGACAGGGAAACGCCCGGTC**CCATTC**CGAACCCGGAAGCTAAGCCTGTC
TGCGCCG**ATGGTACTGC**ACTCGACAGGGTGTGGGAGAGTAGGACACCGCCGAACA

9 116 **7.8**
GTTACGGCGGCCATAGCGACAGGGAAACGCCCGGTC**CCATTC**CGAACCCGGAAGCTAAGCCTGTC
TGCGCCGATGGTACTGCACTCGACAGGGTGTGGGAGAGTAGGACACCGCCGAACA

2

>NR_075455.1 Methanoculleus marisnigri JR1 strain JR1 5S ribosomal RNA, complete sequence
TAGCG**GCCAT**AGCAGAGGGGAAACACCTGGAC**CCATTC**CGAACCCAGCAGTTAAGCCCTCTCACG
TGCCATG**TGGTACTG**AGGTGCGCGAGCCCTCGGGAAGCACGGCTCGCTGCTATTAC

9 117 **7.7**

TAGCGGCCATAGCAGAGGGGAAACACCTGGAC**CCATTC**CGAACCCA**GCAGT**TAAGCCCTCTCACG
TGCCATGTGGTACTGAGGTGCGCGAGCCCTCGGGAAGCACGGCTCGCTGCTATTAC

3

>NZ_LS992663.1 Rickettsia typhi isolate TM2540 chromosome TM2540
CTTGGTGGTCATAGCATGAGTGAAACACACGATCCCATCCCGAACTCGAACGTGAAACCTCATAGC
GCT**AATGGTACT**ATGTCATAAGTCATGGGAGAGTAAGTC**ACTGCCA**AGC

8 111 **7.2**

CTTGGTGGTCATAGCATGAGTGAAACACACGATCCCATCCCGAACTCGAACGTGAAACCTCATAGC
GCT**AATGG**TACTATGTCATAAGTCATGGGAGAGTAAGTCACTGCCAAGC

1

> NR_121856.1 Rhodococcus pyridinivorans 5S ribosomal RNA, complete sequence
TACGGCGGTCATAGCGACAGGGAAACGCCCGGTC**CCATTC**CGAACCCGGAAGCTAAGCCTGTCTG
CGCCG**ATGGTACTGC**ACTCGACAGGGTGTGGGAGAGTAGGACACCGCCGAAC

8 113 **7.1**
TACGGCGGTCATAGCGACAGGGAAACGCCCGGTC**CCATTC**CGAACCCGGAAGCTAAGCCTGTCTG
CGCCGATGGTACTGCACTCGACAGGGTGTGGGAGAGTAGGACACCGCCGAAC

2

>CR848038.1 Chlamydophila abortus strain S26/3, complete genome
GCTTGGTGAT**AATGG**AAAAAGGGATACACCTGATA**CCATTC**CGAACTCAGAAGTTAAGCCTTTTA
TCGCCG**ATGGTACT**ATACA**CAAGA**GTATGGGAGAGTAGGTCGTCGCCAAGCT

8 113 **7.1**

GCTTGGTGAT**AATGG**AAAAAGGGATACACCTGA**TACCATTC**CGAACTCAGAAGTTAAGCCTTTTA
TCGCCGATGGTACTATACACAAGAGTATGGGAGAGTAGGTCGTCGCCAAGCT

5

>NR_075339.1 Methanosphaera stadtmanae DSM 3091 strain DSM 3091 5S ribosomal RNA
TGGTACGGCGACCATAGCGATAGGGTCACATCTGAACTCGTCTCGATCTCAGTAATAAAGTCTATC
CACGTTTTGTT**TGGTACT**AT**AGATGGTA**AGTCTATGGGAATTTCAGAAAG

8 112 **7.1**

TGGTACGGCG**ACCAT**AGCGATAGGGTCA**CATCT**GAACTCGTCTCGATCT**CAGTA**ATAAAGTCTAT
CCACGTTTTGTTTGGTACTATAGATGGTAAGTCTATGGGAATTTCAGAAAG

3

> NR_075227.1 Rhodococcus erythropolis 5S ribosomal RNA gene, complete sequence NCBI
GTTACGGCGGTCATAGCGAAGGGGAAACGCCCGGTC**CCATTC**CGAACCCGGAAGCTAAGCCCTTC
AGCGCCG**ATGGTACTGC**ACTCGACAGGGTGTGGGAGAGTAGGACACCGCCGAACA

8 116 **6.9**

GTTACGGCGGTCATAGCGAAGGGGAAACGCCCGGTC**CCATTC**CGAACCCGGAAGCTAAGCCCTTC
AGCGCCGATGGTACTGCACTCGACAGGGTGTGGGAGAGTAGGACACCGCCGAACA

2

>Rhodococcus globerulus strain 4J2A2, whole genome shotgun sequence NCBI: NZ_CEDU01000055.1 5S

GTTACGGCGGTCATAGCGAAGGGGAAACGCCCGGTCCCATTCCGAACCCGGAAGCTAAGCCCTTC
AGCGCCGATGGTACTGCACTCGACAGGGTGTGGGAGAGTAGGACACCGCCGAACA

8 116 **6.9**
GTTACGGCGGTCATAGCGAAGGGGAAACGCCCGGTCCCATTCCGAACCCGGAAGCTAAGCCCTTC
AGCGCCGATGGTACTGCACTCGACAGGGTGTGGGAGAGTAGGACACCGCCGAACA

2

>NR_076076.1 Methanocaldococcus jannaschii DSM 2661 strain DSM 2661 5S ribosomal RNA
TGGTACGGCGGTCATAGCGGGGGGGCCACACCCGAACCCATCCCGAACTCGGAAGTTAAGCCCCC
CAGCGATGCCCCGAGTACTGCCATCTGGCGGGAAAGGGGCGACACCGCCGGCCAC

8 116 **6.9**

TGGTACGGCGGTCATAGCGGGGGGGCCACACCCGAACCCATCCCGAACTCGGAAGTTAAGCCCCC
CAGCGATGCCCCGAGTACTGCCATCTGGCGGGAAAGGGGCGACACCGCCGGCCAC

2

>NR_075165.1 Nanoarchaeum equitans Kin4-M 5S ribosomal RNA, complete sequence
AGGTTTCGTGGGAGGGCCATAGCGGCCCGGGAACCACCCGTACCCATCTCGAACACGGAAGTTAA
GCCGGGCCGCGTCCCGAGTGGTACTGCCCCGCGAAGGGGTGGGAAGCTCGGGATGCCC

8 119 **6.7**

AGGTTTCGTGGGAGGGCCATAGCGGCCCGGGAACCACCCGTACCCATCTCGAACACGGAAGTTAA
GCCGGGCCGCGTCCCGAGTGGTACTGCCCCGCGAAGGGGTGGGAAGCTCGGGATGCCC

2

>NR_103229.1 Methanosaeta harundinacea 6Ac strain 6Ac 5S ribosomal RNA, complete sequence
CGAGTGTGGCGGCCATAGCGGCGGGGCAACTCCTGAACCCATTCCGAACTCAGAAGATAAGCCC
GCGTGCGTTCCATACTGTACTTAGGTGCGCGAGCCCTCGGGAACTATGGATCGCTGCCA

8 119 **6.7**

CGAGTGTGGCGGCCATAGCGGCGGGGCAACTCCTGAACCCATTCCGAACTCAGAAGATAAGCCCG
CGTGCGTTCCATACTGTACTTAGGTGCGCGAGCCCTCGGGAACTATGGATCGCTGCCA

2

>NR_075285.1 Thermoplasma volcanium GSS1 strain GSS1 5S ribosomal RNA, complete sequence
GGCAACGGTCATAGCAATAGGGAAACACCAGATCCCATTCCGAACTCGACGGTTAAGCCTGTTGC
GTATTACGTTGTACTGTATTCCGCGAGGGTACGGGAAGCGTAGTATGCTGTTGCCATT

8 119 **6.7**

GGCAACGGTCATAGCAATAGGGAAACACCAGATCCCATTCCGAACTCGACGGTTAAGCCTGTTGC
GTATTACGTTGTACTGTATTCCGCGAGGGTACGGGAAGCGTAGTATGCTGTTGCCATT

3

>NR_075125.1 Methanosarcina mazei 5S ribosomal RNA gene, complete sequence
AGTTTGGCGGCCATAGCGGCAGGGCAACTCCTGTACCCATCCCGAACACAGAAGATAAGCCTGCC
CGCGTTCCTTACTGTACTGAAGTGTGCGAGCCTTCGGGAACTCTGGATCGCTGCCAAGCTCACCT

8 126 **6.4**

AGTTTGGCGGCCATAGCGGCAGGGCAACTCCTGTACCCATCCCGAACACAGAAGATAAGCCTGCC
CGCGTTCCTTACTGTACTGAAGTGTGCGAGCCTTCGGGAACTCTGGATCGCTGCCAAGCTCACCT

2

>NR_075338.1 Methanosarcina barkeri str. Fusaro strain Fusaro 5S ribosomal RNA, complete sequence
AGAGTTTGGCGGCCATAGCGGCAGTGTAACTCCTGTACCCATCCCGAACACAGTAGATAAGCCTG
CCCGCGTTCCTTACTGTACTGAAGTGTGCGAGCCTTCGGGAACTCTGGATCGCTGCCATACTCAC

8 127 **6.3**

AGAGTTTGGCGGCCATAGCGGCAGTGTAACTCCTGTACCCATCCCGAACACAGTAGATAAGCCTG
CCCGCGTTCCTTACTGTACTGAAGTGTGCGAGCCTTCGGGAACTCTGGATCGCTGCCATACTCACC

4

>NR_075309.1 Methanococcus maripaludis S2 strain S2 5S ribosomal RNA, complete sequence

CATAACGGTCATAGCGGAGGAGTTACATCCGATCCCATCCCGATCTCGGAAATTAAGCCCTCCTGC GATTCATTAAGTACTGCCATCTGGTGGGAACAATGTGACGCTGTTAGTC

7 111 **6.3**

CATAACGGTCATAGCGGAGGAGTTACATCCGATCCCATCCCGATCTCGGAAATTAAGCCCTCCTGC GATTCATTAAGTACTGCCATCTGGTGGGAACAATGTGACGCTGTTAGTC

4

>NR_075749.1 Methanocaldococcus infernus ME strain ME 5S ribosomal RNA
TACGGCGGCCATAGCGGGGGGGCTACACCCGATCCCATTCCGAACTCGGAAGTTAAGCCCCCCAG CGATGCCCCGAGTACTGCCTTCTGGCGGGAAAGGGGCGACGCCGCCGGCC

7 111 **6.3**

TACGGCGGCCATAGCGGGGGGGCTACACCCGATCCCATTCCGAACTCGGAAGTTAAGCCCCCCAG CGATGCCCCGAGTACTGCCTTCTGGCGGGAAAGGGGCGACGCCGCCGGCC

3

>NR_103265.1 Nanoarchaeum equitans Kin4-M 5S ribosomal RNA, complete sequence
GGAGGGCCATAGCGGCCCGGGAACCACCCGTACCCATCTCGAACACGGAAGTTAAGCCGGGCCGC GTCCCGAGTGGTACTGCCCCGCGAAGGGGTGGGAAGCTCGGGATGCCCTCCC

7 113 **6.2**

GGAGGGCCATAGCGGCCCGGGAACCACCCGTACCCATCTCGAACACGGAAGTTAAGCCGGGCCG CGTCCCGAGTGGTACTGCCCCGCGAAGGGGTGGGAAGCTCGGGATGCCCTCCC

2

>NR_103238.1 Methanothermobacter marburgensis str. Marburg strain Marburg 5S ribosomal RNA
CATGGCGGTCATGGCGCAGGGGTTATACCTGATCTCGTTTCGATCTCAGTAGTTAAGTCCTGCTGCG TTGTGGGTGTGTACTGCGGTGTTTTGCTGTGGGAAGCCCATTTCACTGCCAGCC

7 117 **6**

CATGGCGGTCATGGCGCAGGGGTTATACCTGATCTCGTTTCGATCTCAGTAGTTAAGTCCTGCTGC GTTGTGGGTGTGTACTGCGGTGTTTTGCTGTGGGAAGCCCATTTCACTGCCAGCC

4

>NR_103433.1 Methanolobus psychrophilus R15 strain R15 5S ribosomal RNA, complete sequence
TGGCGGCCATAGCGGCGGGGCAATTCCTGTACCCTTTCCGAACACAGAAGATAAGTCCGCCAGCG TTCCATACTGTACTGAAGTACGCGAGTCTTCGGGAACTACGGATCGCTGCCAGCTC

7 117 **6**

TGGCGGCCATAGCGGCGGGGCAATTCCTGTACCCTTTCCGAACACAGAAGATAAGTCCGCCAGCG TTCCATACTGTACTGAAGTACGCGAGTCTTCGGGAACTACGGATCGCTGCCAGCTC

2

>NR_075663.1 Halomicrobium mukohataei DSM 12286 strain DSM 12286 5S ribosomal RNA
AGGCGGCCATAGCGGCAGGGAAACACCCGTACCCATCCCGAACACGGACGTTAAGCCTGCCAGC GTTGCGGTGAGTACTGGGTGTGCGAACCCCTGGGAAAGCCGGTTCGCCGCCTGCCA

7 117 **5.9**

AGGCGGCCATAGCGGCAGGGAAACACCCGTACCCATCCCGAACACGGACGTTAAGCCTGCCAGC GTTGCGGTGAGTACTGGGGTGTGCGAACCCCTGGGAAAGCCGGTTCGCCGCCTGCCA

3

>NR_075372.1 Methanospirillum hungatei JF-1 strain JF-1 5S ribosomal RNA, complete sequence
AATAGCGGCCACAGCAGGTGTGTCACACCCGTTCCCATTCCGAACACGGAAGTTAAGACACCTCA CGTGGATGACGGTACTGAGGTACGCGAGTCCTCGGGAAATCATCCTCGCTGCTATTG

7 118 **5.9**

AATAGCGGCCACAGCAGGTGTGTCACACCCGTTCCCATTCCGAACACGGAAGTTAAGACACCTCA CGTGGATGACGGTACTGAGGTACGCGAGTCCTCGGGAAATCATCCTCGCTGCTATTG

3

>NR_075189.1 Methanocella arvoryzae MRE50 strain MRE50 5S ribosomal RNA, complete sequence

GTGGGTTTTGGCG**GCCAT**AGCACCAGTGTTACACCCGGTC**CCATTC**CGAACCCGGAAGTTAAGCC
TGGTCACGTAATATGCT**GTACT**AA**GGTAC**GCGAGTCCCTGGGAACCATAATACG**CTGCCA**ACCCA
CCAACAAT

7 134 **5.2**

GTGGGTTTTGGCGGCCATAGCACCAGTGTTACACCCGGTC**CCATTC**CGAACCCGGAAGTTAAGCCT
GGTCACGTAATATGCTGTACTAAGGTACGCGAGTCCCTGGGA**ACCAT**AATACGCTGCCAACCCACC
AACAAT

3
